# Impact of DJ stenting on subacute renal recovery after flexible ureterorenoscopy

**DOI:** 10.1007/s00345-026-06552-5

**Published:** 2026-06-23

**Authors:** Gökhan Ecer, Selim Soytürk, Eren Erol, Abdullah Altunhan, Haider Nihad Izaddin Alalam, Selçuk Güven, Mehmet Balasar

**Affiliations:** 1 Urology Department, Karapınar State Hospital, Konya, Turkey; 2 Urology Department, Necmettin Erbakan University School of Medicine, Konya, Turkey; 3 Urology Department, Akşehir State Hospital, Konya, Turkey

**Keywords:** Flexible ureterorenoscopy, Kidney injury molecule-1, DJ stent, Renal injury, Biomarkers

## Abstract

**Background:**

Routine ureteral stenting after flexible ureterorenoscopy (F-URS) remains controversial. While stents are used to secure drainage, their impact on subclinical renal tubular injury is not well defined. This study aimed to evaluate the effect of postoperative Double-J (DJ) stenting on acute and subacute renal injury using urinary Kidney Injury Molecule-1 (KIM-1) as a sensitive biomarker.

**Methods:**

We conducted a retrospective analysis of a prospectively maintained database including 117 patients who underwent F-URS for renal stones. To minimize selection bias, patients with severe ureteral injury (PULS Grade ≥ 3) were excluded, and a ureteral access sheath (UAS) was utilized in all cases to standardize intrarenal pressure. Patients were stratified into two groups according to the postoperative drainage strategy: Group 1 (DJ stent) and Group 2 (no stent). Urinary KIM-1 levels normalized to creatinine (KIM-1/Cr) were measured preoperatively, at the postoperative 4th hour (acute phase), and on day 14 (subacute phase).

**Results:**

Demographic characteristics and operative trauma scores (PULS) were comparable between the groups. In the acute phase (4th hour), both groups exhibited a significant and similar rise in KIM-1/Cr levels compared to baseline (*p* < 0.001), with no intergroup difference (*p* = 0.748). However, in the subacute phase (day 14), the stented group had significantly lower KIM-1/Cr levels compared to the non-stented group (0.18 vs. 0.36; *p* = 0.001). Additionally, the stone-free rate (SFR) was significantly higher in the stented group (89.8% vs. 65.5%; *p* = 0.002). Postoperative complication rates were similar (*p* = 0.35).

**Conclusion:**

DJ stent placement does not alter the acute biomarker surge associated with surgical stress but is associated with significantly lower KIM-1/Cr levels during the subacute recovery period. This finding suggests that improved drainage, together with more effective stone clearance, may contribute to better tubular recovery in stented patients. Urinary KIM-1 serves as a valuable objective tool for monitoring subclinical renal injury and may guide individualized stenting decisions.

## Introduction

The global prevalence of urolithiasis is increasing, adversely affecting quality of life and imposing a substantial economic burden on healthcare systems. Advances in minimally invasive surgery have established flexible ureterorenoscopy (F-URS) as a standard treatment for renal and proximal ureteral stones, owing to its high success rates and low invasiveness [1].

Despite the general safety of F-URS, complications such as mucosal laceration, perforation, hematuria, urinary tract infection and extrarenal stone migration may occur [2, 3]. Irrigation-driven intrarenal pressure fluctuations during F-URS can result in subclinical, symptom-independent injury to the proximal tubular epithelium. These findings indicate that although endoscopic stone surgery is minimally invasive, it may still be associated with rare but clinically significant complications that warrant careful consideration.

The use of a postoperative double-J (DJ) stent following F-URS remains a subject of ongoing debate. Stent placement can secure urinary drainage, prevent obstruction, and reduce pain. However, its routine use is controversial due to the potential for irritative symptoms, pelvic pain, and deterioration in quality of life [4]. While some advocate that stenting reduces renal injury, others emphasize the associated discomfort and complications. Notably the effect of postoperative stenting on tubular kidney injury after F-URS has not been systematically characterized using objective biomarkers such as urinary kidney injury molecule-1 (KIM-1) [5].

Conventional functional parameters (e.g., serum creatinine, urine output) are insensitive to early tubular injury. In contrast, urinary KIM-1, released from injured proximal tubular epithelium, has emerged as a reliable early indicator of acute kidney injury [6]. Normalizing KIM-1 to urinary creatinine (KIM-1/Cr) minimises bias from interindividual differences in urine dilution, enabling more robust comparisons across time and between groups.

Against this background, we sought to evaluate the effect of postoperative DJ stent placement on renal tubular injury after F-URS by assessing urinary KIM-1 dynamics at both acute (4-h) and subacute (day 14) time points. Our primary objective was to compare day-14 urinary KIM-1/Cr between stented and non-stented patients. We hypothesised that stenting would not materially affect the acute rise in KIM-1/Cr but would be associated with lower KIM-1/Cr at day 14, consistent with reduced intrarenal pressure and facilitated tubular recovery.

## Materials and methods

### Study design and ethical approval

This study was designed as a single-center, retrospective analysis of a prospectively maintained institutional database at the Department of Urology, Necmettin Erbakan University. As part of an ongoing institutional quality improvement protocol aimed at monitoring subclinical renal tubular injury, urinary KIM-1 measurements are routinely obtained in patients undergoing endoscopic renal stone surgery who meet predefined institutional criteria, including preserved baseline renal function (eGFR > 60 mL/min/1.73 m²), absence of active urinary tract infection, and availability of standardized perioperative urine sampling. Consequently, all biomarker data analyzed in this study were obtained from values recorded as part of standard clinical care. The study protocol was approved by the Institutional Research Ethics Committee (Decision No: 2025/6070) and was conducted in accordance with the ethical principles of the Declaration of Helsinki.

### Patient selection

We retrospectively reviewed the medical records of adult patients (aged 18–75 years) who underwent flexible ureterorenoscopy (F-URS) for renal stones between 2019 and 2024. Inclusion criteria were: (1) presence of renal stones confirmed by non-contrast computed tomography (NCCT), (2) preoperative estimated glomerular filtration rate (eGFR) > 60 mL/min/1.73 m², and (3) sterile preoperative urine culture. Exclusion criteria included solitary kidney, congenital renal anomalies, chronic kidney disease (CKD), history of ipsilateral renal surgery, stone surgery within the preceding 3 months, and active urinary tract infection (positive urine culture and clinical symptoms such as fever, dysuria, flank pain, or elevated inflammatory markers). None of the patients included in the study had a preoperative ureteral stent. To minimize selection bias regarding the decision for stenting, patients who sustained severe intraoperative ureteral injury (Post-Ureteroscopic Lesion Scale Grade ≥ 3) were excluded from the analysis [2]. Also who could not be successfully matched during the PSM process were excluded from matched analyses. Patients in whom ureteral access sheath insertion failed despite gentle manipulation were not included in the study and were managed with temporary DJ stenting followed by delayed re-intervention. Patients were stratified into two groups based on the postoperative drainage strategy: Group 1 (DJ Stent) and Group 2 (No Stent).

### Surgical technique

All procedures were performed under general anesthesia in the lithotomy position by a single experienced surgeon. Following diagnostic cystoscopy and guidewire placement, a ureteral access sheath (UAS) (11/13 Fr, Boston Scientific Navigator™, USA) was utilized in all cases to facilitate instrument passage and maintain low intrarenal pressure. Flexible ureterorenoscopy was performed using a digital or fiber-optic ureteroscope (Flex-X2, Karl Storz™, Germany), and stones were fragmented using a Holmium: YAG laser (200 μm fiber; settings: 10 Hz, 2–2.5 J). The irrigation fluid column was maintained at a constant height of 60 cm in all procedures. The decision to place a postoperative DJ stent was based on the surgeon’s intraoperative assessment of findings such as mucosal edema, minor trauma (PULS Grade 1–2), or potential residual fragment burden. In Group 1, stents were removed on postoperative day 14.

### Data collection and outcome measures

Demographic characteristics, perioperative variables, and laboratory data were extracted from the institutional medical records. The primary outcome was the difference in urinary KIM-1/creatinine (KIM-1/Cr) levels between the groups on postoperative day 14. Secondary outcomes included the acute phase response, assessed by comparing KIM-1/Cr levels at the postoperative 4th hour; postoperative complications recorded according to the Clavien Dindo classification; and the stone-free rate (SFR). SFR was defined as the complete absence of stones or the presence of clinically insignificant residual fragments (< 4 mm) on follow-up NCCT or ultrasonography performed 2–4 weeks postoperatively.

### Biomarker measurement

At our institution, which has extensive experience in renal injury biomarkers and has previously published studies on this topic, urinary KIM-1 measurements are obtained in patients undergoing endoscopic stone surgery. Therefore, during routine perioperative laboratory evaluations and follow-up assessments, urine samples for KIM-1 analysis are systematically collected at predefined time points (preoperative and postoperative).

Urine samples were collected at three specific time points: (1) Preoperative (baseline), (2) Postoperative 4th hour (acute phase), and (3) Postoperative Day 14 (subacute phase). Crucially, the day 14 samples were obtained immediately prior to stent removal or follow-up cystoscopy to avoid procedure-related biomarker elevation. To obtain standardized urine samples, a urethral catheter was placed in all patients at the end of the procedure. For the postoperative 4th hour measurement, the catheter was temporarily clamped for 10 min to allow urine accumulation in the bladder. Subsequently, the urine sample was collected through the catheter. Urinary KIM-1 levels were quantified using commercial enzyme-linked immunosorbent assay (ELISA) kits (Elabscience™, USA). To correct for variations in urine concentration, KIM-1 values were normalized to urinary creatinine and expressed as the KIM-1/Cr ratio.

### Propensity score matching

To reduce selection bias related to non-random DJ stent placement, propensity score matching (PSM) was performed. A propensity score for DJ stent placement was estimated using a logistic regression model including age, sex, comorbidities, hydronephrosis, stone side, stone location, stone size, number of stones, Hounsfield unit, operative time, and PULS grade. Patients were matched 1:1 using nearest neighbor matching without replacement with a caliper of 0.2 of the standard deviation of the logit of the propensity score. Balance between groups was assessed using standardized mean differences (SMD), with an SMD < 0.10 considered acceptable. PSM produced 58 matched pairs; one DJ patient was unmatched and excluded from matched analyses.

### Statistical analysis

Statistical analyses were performed using IBM SPSS Statistics for Windows, version 25.0 (IBM Corp., Armonk, NY, USA). The normality of continuous variables was assessed using the Shapiro-Wilk test. Normally distributed variables were presented as mean ± standard deviation (SD) and compared using the Independent Samples t test. Non-normally distributed variables (including KIM-1 levels) were presented as median (interquartile range) and analyzed using the Mann–Whitney U test. Categorical variables were compared using the Chi-square test or Fisher’s exact test. To minimize selection bias related to non-random postoperative DJ stent placement, propensity score matching (PSM) was performed. A p value < 0.05 was considered statistically significant.

## Results

A total of 117 patients who underwent flexible ureteroscopy were included in the study. Postoperatively, 59 patients received a DJ stent, while 58 patients did not. There were no significant differences between the groups regarding gender distribution, comorbidities, presence of hydronephrosis, or stone location (*p* > 0.05). Primary analyses were conducted in the full cohort (*n* = 117). A sensitivity analysis using propensity score–matched pairs (*n* = 116) yielded consistent results.

Evaluation of PULS scores showed that most patients in both groups had Grade 0 or Grade 1 lesions, with no significant difference between the groups (*p* = 0.99). Postoperative complication rates and the incidence of urinary tract infections were also comparable (*p* = 0.35 and *p* = 0.28, respectively). The stone-free rate was significantly higher in the DJ group (89.8%) than in the non-DJ group (65.5%) (*p* = 0.002).

There were no statistically significant differences between the groups in terms of age, preoperative hemoglobin, or postoperative hemoglobin levels (*p* > 0.05). The number of stones, Hounsfield unit values, and stone size were also comparable between the groups (*p* > 0.05) (Table 1).

**Table 1:** Comparison of demographic, clinical, stone characteristics and perioperative outcomes between groups

	Group 1 (DJ Group) *N*: 59	Group 2 (non-DJ Group) *N*: 58	*P* value
*Gender*			
Male	41 (%69.5)	35 (%60.3)	0.3*
Female	18 (%30.5)	23 (%39.7)	
*Additional disease*			
No disease	36 (%61)	29 (%50)	
Hypertension	6 (%10.2)	7 (%12.1)	
Diabetes Mellitus	6 (%10.2)	4 (%6.9)	0.43*
Other	6 (%10.2)	13 (%22.4)	
HT + DM	5 (%8.5)	5 (%8.6)	
*Hydronephrosis presence*			
Yes	26 (%44.1)	24 (%41.4)	0.76*
No	33 (%55.9)	34 (%58.6)	
*Stone location*			
Upper calyx	5 (%8.5)	4 (%6.9)	
Middle calyx	12 (%20.3)	8 (%13.8)	
Lower calyx	14 (%23.7)	11 (%19)	
Pelvis	10 (%16.9)	22 (%37.9)	
Multiple	18 (%30.5)	13 (%22.4)	0.16*
*PULS grade*			
0	47 (%79.7)	46 (%79.3)	0.99*
1	9 (%15.3)	9 (%15.5)	
2	3 (%5.1)	3 (%5.2)	
3	0	0	
4	0	0	
5	0	0	
*Postop complication*			
0	52 (%88.1)	54 (%93.1)	
1	7 (%11.9)	4 (%6.9)	
2	0	0	
3	0	0	
4	0	0	
5	0	0	0.35*
*Postoperative urinary infection*			
Yes	7 (% 11.9)	11(% 19)	0.28*
No	52 (% 88.1)	47 (% 81)	
*Stone free status*			
Yes	53 (%89.8)	38 (%65.5)	0.002*
No	6 (%10.2)	20 (%34.5)	
*Stone side*			
Right	38 (%64.4)	27 (%46.6)	0.052*
Left	21 (%35.6)	31 (%53.4)	
Age(years ± SD)	48.4 ± 13.9	52.6 ± 12.4	0.09^t^
Preoperative Hemoglobin (g/dL ± SD)	14.1 ± 1.7	13.4 ± 1.9	0.44^t^
Postoperative Hemoglobin (g/dL ± SD)	14.1 ± 1.7	15.3 ± 1.7	0.58^t^
Number of stones	1.41 ± 0.69	1.43 ± 0.65	0.68^a^
Hounsfield unit value	1071 ± 210	974 ± 294	0.41^a^
Stone size (cm ± SD)	14.3 ± 5.4	15.5 ± 4.3	0.092^a^

Bold values indicate statistical significance

*Chi-square test, ^t^ independent t test

^a^Mann–Whitney U test

**Fig. 1:**
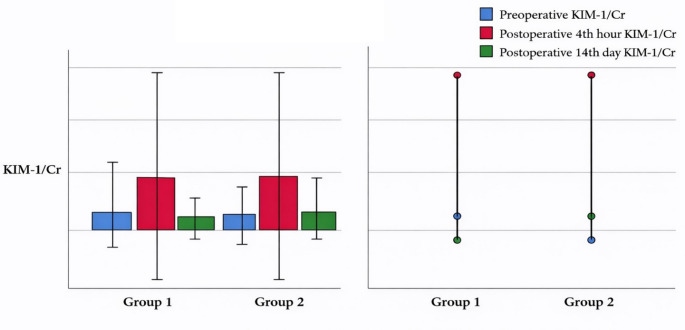
Urinary KIM-1/Cr levels in the DJ stent (Group 1) and non-DJ stent (Group 2) groups measured preoperatively, at the postoperative 4th hour, and on postoperative day 14. Left panel: grouped bar plot with error bars. Right panel: summary marker plot of KIM-1/Cr measurements

In the biomarker analysis, preoperative KIM-1/Cr levels did not differ significantly between the groups (DJ: 0.33; non-DJ: 0.31; *p* = 0.97). At the 4th postoperative hour, both groups showed a marked increase in KIM-1/Cr levels, but the difference between them was not significant (DJ: 0.93; non-DJ: 0.94; *p* = 0.748). By postoperative day 14, KIM-1/Cr levels were significantly lower in the DJ group compared with the non-DJ group (DJ: 0.18; non-DJ: 0.36; *p* = 0.001) (Table 2; Fig. 1).

**Table 2:** Comparison of urinary KIM-1/Cr and serum creatinine levels between the groups

	Group 1 (DJ Group) *N*: 59	Group 2 (non-DJ Group) *N*: 58	*P* value
Preoperative KIM-1/Cr, Median (IQR)	0.33 (0.21)	0.31 (0.21)	0.97^a^
Preoperative serum creatinine level(mg/dl), (min-max)	0.83 (0.67–1.1)	0.87 (0.66–1.2)	0.48^a^
Postoperative 4th hour KIM-1/Cr, Median (IQR)	0.93 (0.63)	0.94 (0.67)	0.748^a^
Postoperative 4th hour serum creatinine (mg/dl), (min-max)	0.88 (0.71–1.2)	0.93 (0.62–1.1)	0.50^a^
Postoperative 14th day KIM-1/Cr, Median (IQR)	0.18 (0.11)	0.36 (0.27)	**0.001** ^a^
Postoperative 14th day serum creatinine (mg/dl), (min-max)	0.85 (0.59–1.1)	0.86 (0.62–1.1)	0.82^a^

Bold values indicate statistical significance

^a^Mann–Whitney U test

In multivariate linear regression analysis adjusting for SFR, stone size, hydronephrosis, and PULS grade, DJ stent placement remained an independent predictor of lower postoperative Day 14 KIM-1 levels (β = − 0.353, *p* < 0.001), whereas SFR was not independently associated with KIM-1 levels.

## Discussion

In this study, postoperative DJ stenting was associated with significantly lower urinary KIM-1 levels on postoperative day 14, while no difference was observed during the early postoperative period. These findings suggest that DJ stenting does not prevent the immediate tubular stress caused by surgical manipulation but may facilitate renal recovery during the subacute phase.

KIM-1 has been recognized as a sensitive biomarker of proximal tubular injury. Unlike conventional renal function parameters such as serum creatinine, which often remain unchanged in mild or early injury, urinary KIM-1 can detect subclinical tubular damage and provide insight into renal recovery processes. KIM-1 levels typically rise shortly after tubular injury, peak within the first few days, and gradually decline during recovery; however, detectable expression may persist up to 14 days, reflecting ongoing tubular repair [7]. Therefore, the day 14 measurement in our study was intended to represent the subacute recovery phase rather than the acute injury peak.

The observation of significantly higher stone free rates in the stented group, together with comparable complication rates, suggests that the protective effect of the stent may primarily be related to improved drainage and reduction of intrarenal pressure rather than fragment clearance. Importantly clinical relevance lies not in the absolute numerical difference, but in the persistence of biomarker elevation. Higher KIM-1 levels on postoperative day 14 in the non-stented group suggest ongoing subclinical tubular stress rather than a transient surgical effect. Even when serum creatinine remains unchanged, sustained elevation of KIM-1 may indicate continued tubular injury.

The literature remains inconclusive regarding the routine use of DJ stents after F-URS. Some studies suggest that stent placement helps prevent obstruction, reduces postoperative pain, and improves stone-free rates [4, 8]. Conversely, others have reported that DJ stents are associated with adverse effects such as dysuria, urinary frequency, hematuria, and a decline in overall quality of life [9]. It should be emphasized that KIM-1 measurements were obtained preoperatively, at the postoperative 4th hour, and on postoperative day 14; therefore, biomarker levels did not influence the intraoperative decision to place a DJ stent, which was based solely on surgical findings.

Serum creatinine and urine output are insufficient for detecting acute tubular injury in its early stages [10]. In recent years, KIM-1 has emerged as a reliable biomarker of acute kidney injury, as its urinary levels rise in response to proximal tubular epithelial damage [6]. Previous clinical studies have demonstrated a significant increase in KIM-1 levels following endoscopic stone surgery, attributing this rise to the elevation of intrarenal pressure during the procedure [11]. Our findings align with these observations while further elucidating how DJ stenting influences renal injury when assessed via this damage biomarker. Qian et al. reported that in patients with unilateral ureteral obstruction, urinary KIM-1 levels declined significantly after DJ stent placement, whereas no such reduction was observed in non-stented patients [12]. In our cohort, however, our study uniquely demonstrates how this pattern manifests in patients undergoing F-URS, thereby providing a novel contribution to the current literature.

In our study, the absence of a significant difference in KIM-1 levels between the groups at the 4th postoperative hour suggests that DJ stenting has a limited influence on acute tubular injury in the immediate postoperative phase. This interpretation is corroborated by previous reports showing that serum creatinine can transiently increase during the early days following F-URS, reflecting the acute physiological response to the procedure [13]. Consistent with this, our cohort exhibited a universal increase in KIM-1 levels in the acute period compared to preoperative baselines. However, the significantly lower KIM-1 levels observed in the DJ group by postoperative day 14 suggest that stenting mitigates subacute renal injury, potentially by alleviating intrarenal pressure and supporting tubular recovery. This finding indicates that the clinical utility of a DJ stent extends beyond simple mechanical drainage, possibly actively contributing to the renal healing process.

Previous studies have demonstrated that DJ stents can enhance postoperative fragment clearance by preventing transient ureteral obstruction and reducing intrarenal pressure after ureteroscopic stone surgery [14]. Improved drainage may, in turn, mitigate sustained tubular stress and promote renal recovery. Accordingly, the lower KIM-1 levels observed on postoperative day 14 in the DJ group likely reflect not only reduced intrarenal pressure but also superior stone clearance, supporting a combined mechanical and biological protective effect of DJ stenting on subacute renal tubular injury. The higher stone free rate observed in the DJ stent group may have contributed to the lower postoperative Day 14 KIM-1 levels by reducing persistent obstruction, residual fragment-related irritation, and ongoing tubular stress. Recent evidence suggests that even small residual stone fragments may have biological and clinical significance and may contribute to stone-related events and the need for reintervention [15]. Improved drainage and facilitated fragment clearance may therefore represent important mechanisms underlying the observed biomarker differences between the groups. Although the higher stone free rate in the DJ group could theoretically confound the interpretation of subacute KIM-1 differences, multivariate analysis adjusting for SFR demonstrated that DJ stent placement remained an independent predictor of lower postoperative Day 14 KIM-1 levels, whereas SFR itself was not independently associated with biomarker levels. These findings suggest that the reduction in KIM-1 among stented patients cannot be explained solely by improved stone clearance and likely reflects an additional protective effect related to enhanced drainage and tubular recovery. Nevertheless, our findings do not support universal stenting after F-URS. Instead, postoperative stent placement should be individualized, and biomarkers such as KIM-1 may help guide decision-making in selected patients.

The impact of stenting on postoperative morbidity remains debated. While some multicenter data suggest a protective role against complications [16], systematic reviews indicate that stenting offers no significant reduction in overall complication rates and may exacerbate irritative symptoms [17]. In our cohort, complication rates were statistically comparable between the stented and non-stented groups (*p* = 0.35). These findings align with the growing consensus that stent omission after uncomplicated F-URS is safe and does not compromise postoperative safety profiles.

A persistent clinical challenge in endourology is determining which patients truly benefit from postoperative DJ stenting. Current clinical decision making relies heavily on subjective intraoperative assessments, such as stone burden, edema, and mucosal integrity; however, it is well recognized that these macroscopic parameters do not fully reflect the microscopic renal trauma that may develop post procedure [18]. In this context, KIM-1 has emerged as a vital candidate to bridge this clinical gap, serving as an early and sensitive indicator of proximal tubular injury [6]. The persistence of elevated KIM-1 levels on postoperative day 14 in non-stented patients suggests that a biomarker-based approach may be essential, particularly when conventional clinical indicators fail to capture ongoing subclinical renal stress.

Mechanistically, the intrarenal pressure fluctuations and mucosal trauma inherent to F-URS can lead to prolonged tubular stress and delayed healing if adequate drainage is not maintained. Qian et al. demonstrated that following the relief of obstruction, urinary KIM-1 levels normalized significantly faster in stented patients compared to those without stents [12]. This aligns with our observation that the DJ stent functions not merely as a mechanical drainage device, but potentially as a facilitator of the tubular recovery process.

A critical insight from our study is the divergence in recovery trajectories: although both groups exhibited an identical acute stress response, biochemical stress persisted significantly longer in the non-stented group. This pattern implies that omitting a stent may expose certain patients to unrecognized subacute tubular injury. While current guidelines deem stent omission safe in “uncomplicated” cases, these recommendations lack support from biomarker-based data [19]. Our results suggest that the definition of “uncomplicated URS” may need to be refined from a biochemical perspective. Such a reassessment could help identify specific patient subsets likely those with borderline stone burden or subclinical edema who would derive functional benefit from postoperative stenting.

An important strength of the present study is the use of propensity score matching to account for potential confounding associated with non-random DJ stent placement. This approach enhances the validity of our findings by reducing selection bias inherent to retrospective analyses. Similar methodology has been successfully applied in contemporary endourological studies to balance baseline characteristics and improve causal inference when randomization is not feasible, particularly in studies evaluating surgical techniques and perioperative outcomes after RIRS [20].

In contrast to urinary KIM-1 levels, serum creatinine did not differ significantly between the groups during follow-up. This suggests that the higher KIM-1 levels in the non-stented group reflect subclinical tubular stress rather than overt renal dysfunction. Serum creatinine is relatively insensitive for detecting early renal injury, whereas KIM-1 is a more sensitive biomarker of acute and subacute tubular damage.

### Limitations

Certain limitations should be considered when interpreting these results. The retrospective, single-center design and the lack of randomization for stent placement introduce potential selection bias. Although the demographic characteristics and, crucially, the PULS scores were similar between the groups, a causal relationship cannot be definitively established. Comorbidities were recorded descriptively rather than using a validated comorbidity index which may limit the standardization of comorbidity assessment. Additionally, the absence of long-term (≥ 3 months) follow-up data on renal function and KIM-1/Cr levels limits our ability to determine the clinical permanence of the subacute differences observed.

## Conclusion

Postoperative DJ stenting after flexible ureteroscopy was associated with lower urinary KIM-1 levels in the subacute period, suggesting a potential protective effect on renal tubular recovery. However, these findings do not support universal stenting for all patients undergoing uncomplicated procedures. Although preoperative decision making cannot be inferred from the present study, urinary KIM-1 may represent a promising biomarker for future individualized stenting strategies.

## Data Availability

The datasets used and/or analyzed during the current study are available from the corresponding author upon reasonable request.
